# New Models and Insights into Primate Evolutionary Morphology

**DOI:** 10.1155/2011/895175

**Published:** 2011-12-01

**Authors:** Anne M. Burrows, Kathleen M. Muldoon, Adam D. Sylvester

**Affiliations:** ^1^Department of Physical Therapy, Duquesne University, 600 Forbes Avenue, Pittsburgh, PA 15282, USA; ^2^Department of Anthropology, University of Pittsburgh, Pittsburgh, PA 15260, USA; ^3^Department of Anatomy, Dartmouth Medical School, HB 7100, Hanover, NH 03743, USA; ^4^Department of Anthropology, Dartmouth College, HB 6047, Hanover, NH 03743, USA; ^5^Department of Human Evolution, Max Planck Institute for Evolutionary Anthropology, Deutscher Platz 6, Leipzig D-04103, Germany

This special issue highlights current research by emerging leaders in the fields of functional and evolutionary anatomy. Evolutionary morphology is concerned with the ways in which anatomical structures respond to environmental pressures, both within the lifetime of an individual and over geological time. Evolutionary morphology as a discipline is focused on understanding the factors influential in primate evolution and evolutionary innovations. These considerations are reflected in this special issue with authors' contributions drawn from diverse areas including comparative and functional anatomy, evolutionary biology, neurobiology, biomechanics, and growth and development. 

Several papers in this special issue focus on the anatomical study of adaptation by focusing on the functional morphology of feeding. M. N. Muchlinski (University of Kentucky) and J. M. G. Perry (Midwestern University) investigate adaptations to nectar feeding in the skull of strepsirrhines (the clade including lemurs and lorises). These authors find that strepsirrhine nectar feeders have elongated midfaces and suggest that these primates are cross-pollinators that played a key role in the coevolution of primates and flowering plants. 

An examination of the musculature related to feeding was carried out by J. M. G. Perry and colleagues (Midwestern University) in select strepsirrhine species. These authors analyzed physiological cross-sectional area of masticatory muscles in order to estimate the potential bite force a particular species could generate in living primates which can, in turn, inform our understanding of food processing behaviors in extinct primates represented in the fossil record. 

Tying together the study of feeding behavior and body size, A. Hartstone-Rose (Penn State University, Altoona) and J. M. G. Perry (Midwestern University) investigate the relationship between maximum ingested food size and body size. These authors previously found that there is a strong scaling relationship between these two variables within species of strepsirrhine primates but their present study shows a lack of scaling relationships *within* strepsirrhine species. This study has important implications for the study of scaling questions in general. 

Four papers in this special issue focus on the issues of growth and development in primate evolutionary morphology. T. Ito and colleagues (Primate Research Institute, Kyoto University) tackle the issue of macaque evolutionary history by examining allometry of the bony face in two closely related macaque species. Macaques present a range of interspecific body sizes, which itself can cause confusion when trying to interpret evolution of morphological features among species. Results of their present study demonstrate that some craniofacial morphologies in macaques are the result of differences in body size alone while others seem to be genuinely adaptive, giving these features greater weight in phylogenetic analyses. 

Emphasis on the skull continues with an investigation into the relationship between the growing mandible and permanent molars in baboons and apes by J. C. Boughner (University of Saskatchewan). During the transition from deciduous to permanent dentition, increased space for molars must be created in the mandible and this growth and development relationship must be timed properly. J. C. Boughner finds that there are different rates of mandibular growth and shape development between baboons and apes from infancy through adulthood. These results highlight the strong selective pressures among timing of growth and development of the dentition and the mandible and how tight this relationship needs to be. 

Moving to the growth and development of the entire body plan, D. R. Bolter (Modesto College) compares growth and development of crested langurs, arboreal monkeys, with that of vervet monkeys, terrestrial animals. Using data from limb growth, brain growth, and body mass, this author finds that growth trajectories differ markedly between these species throughout ontogeny. These findings highlight the complex interplay among diet, phylogeny, ecology, and locomotion in the evolution of growth and development. 

Three papers in this special issue examine adaptive morphologies of primate locomotion. The primate lower limb has long been the focus of investigations in evolutionary morphology. J. J. Baker and colleagues (Slippery Rock University) examine the relationship between growth and development lower limb musculature relative to locomotor style in strepsirrhine species. These authors find that infant strepsirrhines show no difference in proximal-distal distribution of muscle mass between generalized arboreal quadrupeds versus leapers, but adult specimens show discrete differences in proximal-distal distributions. These results suggest that ontogenetic development of proximal-distal lower limb muscle mass distributions may be especially important in primates with specialized locomotor modes. 

Further exploration of lower limbs comes from J. B. Hanna (West Virginia School of Osteopathic Medicine) and D. Schmitt (and Duke University) who synthesize the literature base on triceps surae muscle group across primate taxa into a review of variation in fiber type distribution and muscle mass. These authors show that the triceps surae group is similar among the distantly related great apes, atelines, and lorises, and they relate this morphological similarity to the similar locomotor style that they all use, vertical climbing. These results are especially interesting in light of the potential evolutionary tie between vertical climbing and bipedalism.

Osteological correlates of lower limb locomotor behavior are explored by K. J. Weinstein (Dickinson College) by using variety of macaque species. Macaques, like humans, occupy a wide range of altitudes and climates, and the author investigates the relationship among these ecological variables and lower limb morphology. Her results show that high elevations and temperature climates are associated with short lower limbs while low elevations and tropical climates are associated with long lower limbs. These results lend support to the influence of ecological factors on evolution of lower limb morphology.

Primate brain evolution has been one of the dominant research themes in primate evolutionary morphology. Whether evolution of specific parts of the brain is adaptive or more the result of general scaling principles has been debated. S. D. Dobson (Dartmouth College) and C. C. Sherwood (The George Washington University) explore this debate by examining volumes of the facial motor nucleus in brains of catarrhine primates. These authors demonstrate that volume of this nucleus is not correlated with volume of other motor nuclei, suggesting that behavioral and ecological factors may be important in its evolution. Results of this investigation have potentially important implications for the ability of brainstem motor nuclei to evolve independent of other developmentally linked structures, the notion of the “mosaic” evolution of the primate brain.

The development and use of nonprimate animal models has provided valuable insight into many areas of primate evolution, human growth and development, and the processual events associated with them. S. J. Rehorek and colleagues (Slippery Rock University) evaluate the utility of a laboratory rabbit model to understanding the development of the lacrimal region and nasolacrimal duct of humans. The nasolacrimal duct connects the orbital and nasal cavities, two key areas both in primate evolution and human growth and development. These authors use three-dimensional reconstruction of serially sectioned juvenile rabbits to depict the nasolacrimal duct course. While they find some similarities between rabbits and haplorhine primates, the authors ultimately conclude that there is poor congruity between nasolacrimal duct morphology in rabbits and humans. This result bears great significance as it indicates that rabbits are a poor choice for an animal model of the human lacrimal region in general. 

The 11 articles in this special issue cover a wide range of topics in the field of primate evolutionary morphology. The diversity of topics in this special issue reflects the breadth of interests of emerging evolutionary morphologists. This field of research is rapidly expanding and providing answers to many intriguing questions focused on primate evolution. Our ultimate hope for this special issue is that it serves as a stimulus for further research into primate evolutionary morphology. 

##  Acknowledgments

We are enormously grateful to the contributing authors for their scholarly contributions to the study of primate evolutionary morphology. We also offer our sincere thanks to the many reviewers who gave so generously of their time and thoughts on each paper.

*Anne M. Burrows* *Kathleen M. Muldoon* *Adam D. Sylvester*

